# In Vitro and In Vivo Studies of Heraclenol as a Novel Bacterial Histidine Biosynthesis Inhibitor against Invasive and Biofilm-Forming Uropathogenic *Escherichia coli*

**DOI:** 10.3390/antibiotics12010110

**Published:** 2023-01-06

**Authors:** Harpreet Kaur, Naveen Chaudhary, Vinay Modgil, Manmohit Kalia, Vishal Kant, Balvinder Mohan, Alka Bhatia, Neelam Taneja

**Affiliations:** 1 Department of Medical Microbiology, Postgraduate Institute of Medical Education and Research, Chandigarh 160012, India; 2 Department of Biology, State University of New York, Binghamton, NY 13902, USA; 3 Department of Experimental Medicine and Biotechnology, Postgraduate Institute of Medical Education and Research, Chandigarh 160012, India

**Keywords:** urinary tract infections, multidrug resistance, HisC, heraclenol, in vitro, in vivo

## Abstract

Globally, urinary tract infections (UTIs) are one of the most frequent bacterial infections. Uropathogenic *Escherichia coli* (UPEC) are the predominant etiological agents causing community and healthcare-associated UTIs. Biofilm formation is an important pathogenetic mechanism of UPEC responsible for chronic and recurrent infections. The development of high levels of antimicrobial resistance (AMR) among UPEC has complicated therapeutic management. Newer antimicrobial agents are needed to tackle the increasing trend of AMR and inhibit biofilms. Heraclenol is a natural furocoumarin compound that inhibits histidine biosynthesis selectively. In this study, for the first time, we have demonstrated the antimicrobial and antibiofilm activity of heraclenol against UPEC. The drug reduced the bacterial load in the murine catheter UTI model by ≥4 logs. The drug effectively reduced bacterial loads in kidney, bladder, and urine samples. On histopathological examination, heraclenol treatment showed a reversal of inflammatory changes in the bladder and kidney tissues. It reduced the biofilm formation by 70%. The MIC value of heraclenol was observed to be high (1024 µg/mL), though the drug at MIC concentration did not have significant cytotoxicity on the *Vero* cell line. Further molecular docking revealed that heraclenol binds to the active site of the HisC, thereby preventing its activation by native substrate, which might be responsible for its antibacterial and antibiofilm activity. Since the high MIC of heraclenol is not achievable clinically in human tissues, further chemical modifications will be required to lower the drug’s MIC value and increase its potency. Alternatively, its synergistic action with other antimicrobials may also be studied.

## 1. Introduction

Urinary tract infections (UTIs) are one of the most common infections globally, responsible for 8.3 million visits to outpatient clinics, 1 million visits to emergency departments, and 1,000,000 hospitalizations worldwide [1]. It is anticipated that 40% of women will have at least one UTI in their lives, and 11% of women over the age of 18 will have a UTI episode each year [2]. UTIs are caused by a wide range of bacteria, uropathogenic *E.coli* (UPEC) being the most frequently identified causative organism in both uncomplicated and complicated UTIs [3]. UPEC is responsible for 90% of all UTIs in ambulatory patients and up to 50% of all nosocomial UTIs [1]. UPEC harbor unique virulence characteristics, such as type 1 pili and iron-chelating factors (siderophores), which enable them to colonize the human mucosal surface and overcome host defenses, allowing an invasion of the urinary tract [4]. 

An important pathogenetic mechanism of UPEC is their potential to form biofilms on catheters, responsible for the majority of catheter-associated UTIs [5]. UPEC also forms biofilms inside the uroepithelial cells, known as intracellular biofilm communities (IBCs), which permit the bacteria to survive host immune responses and establish a dormant reservoir leading to relapses of UTIs [6,7]. The treatment of UTIs is becoming more difficult as UPEC have developed resistance to several antibiotic classes, including third-generation cephalosporins, aminoglycosides, fluoroquinolones, and carbapenems, as well as colistin [8]. As a result, new therapeutic targets and newer therapies are urgently needed to combat the rising trend of antibiotic resistance. Strategies to develop new antibacterial drugs that target biosynthetic pathways rather than bacterial growth appear attractive, especially those targeting the metabolic processes, amino acid biosynthesis, and virulome [9,10]. The role of histidine in promoting virulence has been well-studied in *Burkholderia pseudomallei* and *Salmonella typhimurium* [11,12]. HisB enzyme, which catalyzes the conversion of L-histidinol phosphate to L-histidinol, and the L-histidinol dehydrogenase enzyme (HDH), which catalyzes the final reaction of the histidine biosynthesis, have been used as drug targets previously [13]. Based on our earlier work using subtractive genomics and protein interaction network analysis, we identified HisC as a novel drug target for UPEC [10]. In another study, we screened the FDA-approved drug library against HisC, which catalyzes the conversion of imidazole-acetol-phosphate to histidinol-phosphate, an important reaction involved in the biosynthesis of histidine [14]. We found that docetaxel could be repurposed as a potential antimicrobial agent against UPEC [14]. The enzymes of the histidine biosynthesis pathway provide a selective target as human microflora do not have this pathway, thus mitigating the collateral damage to human microflora [10,15]. With the increase in drug-resistant microorganisms and the paucity of newer antimicrobial agents, natural extracts and natural-product-based antibiofilm agents may serve as an important source of new drugs or templates for the development of new synthetic drugs [16,17]. Heraclenol is a coumarin derivative isolated from the roots of *Angelica Lucida*, *Ducrosia anethfolia*, and *Prangos pabularia* [18]. In a previous study, we carried out in silico screening of natural compounds taken from the ZINC database against HisC (target protein) and found heraclenol to be one of the best inhibitors [19]. In the present study, we tested the efficacy of heraclenol in clearing UPEC infections in an in vivo murine transurethral catheter UTI model and also demonstrated its antibiofilm activity. We further studied the mechanism of action of heraclenol against histidinol-phospho aminotransferase (HisC) using molecular docking. For the first time, we have demonstrated that heraclenol could be a potential new antimicrobial agent effective against invasive and biofilm-forming UPEC, which are an emergent challenge to be treated [8].

## 2. Materials and Methods

**Figure 1:**
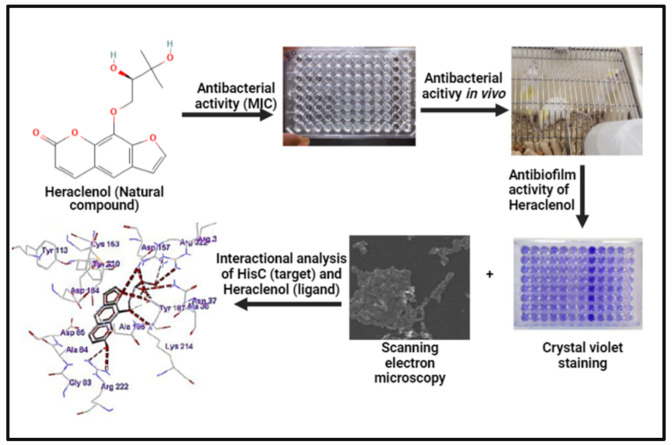
Graphical representation of the methodology of the present study.

Figure 1 depicts the overall flow of the methodology used in this study.

### 2.1. Bacterial Challenge Organisms

We used a highly virulent UPEC strain CFT073 (Microbial Type Culture Collection, MTCC 4296) belonging to serotype O6:K2:H1. For biofilm inhibition experiments, we used biofilm-forming *E. coli* MTCC 739 as a reference strain. Both strains were purchased from the Council of Scientific and Industrial Research (CSIR) Institute of Microbial Technology, Chandigarh. The drug heraclenol was purchased from Sigma-Aldrich, India. 

### 2.2. Minimum Inhibitory Concentration (MIC)

The MIC of heraclenol was determined by using the micro broth dilution method, according to Clinical & Laboratory Standards Institute (CLSI) guidelines [20]. A stock solution of heraclenol (2048 µg/mL) was prepared and serially diluted in 100 µL of Muller Hinton Broth (MHB), making concentrations that ranged from 2048 µg/mL to 2 µg/mL in a ninety-six well microtiter plate. Each well received 5 µL of bacterial culture containing 10^5^ CFU/mL. As a positive control, a well containing only MHB was used, and a well containing only drug was taken as a negative control. The plate was incubated for 18 h at 37 °C. The MIC value was determined as the lowest concentration of heraclenol at which no visible growth appeared [21].

### 2.3. Cell Cytotoxicity Analysis Using MTT

To evaluate non-toxic concentrations of the heraclenol, a cell viability assay was performed in a *Vero* monkey kidney cell line using MTT (3-(4, 5-dimethyl thiazolyl) 2, 5-diphenyl-tetrazolium bromide). Cells were seeded in a 96-well flat-bottom microtiter plate at a density of 1 × 10^4^ cells/well and allowed to adhere for 24 h at 37 °C in a carbon dioxide (CO_2_) incubator. After 24 h of incubation, the culture medium was replaced with a fresh medium. Cells were then treated with various concentrations of heraclenol for 24 h at 37 °C in a CO_2_ incubator. After 24 h of incubation, the culture medium was replaced with a fresh medium. Subsequently, 10 μL of MTT solution (5 mg/mL in phosphate-buffered solution) was added to each well, and the plate was incubated for 4 h at 37 °C in a CO_2_ incubator. The medium was then aspirated and the formed formazan crystals were solubilized by adding 50 μL of dimethyl sulfoxide (DMSO) per well for 30 min at 37 °C in a CO_2_ incubator. Finally, the intensity of the dissolved formazan crystals (purple color) was quantified using the microplate reader at 540 nm. The results were expressed as the percentage viability of each sample compared to the negative control (phosphate-buffered saline (PBS) buffer, pH 7.4) [22].

### 2.4. In Vivo Assessment of the Efficacy of Heraclenol

For efficacy testing, five to six-week-old female BALB/c mice were used and kept at the institute’s animal facility. All experiments were performed after ethical clearance (Ref. No.93/IAEC/649) and according to the guidelines of the Animal Ethics Committee (AEC) of the Postgraduate Institute of Medical Education and Research (PGIMER), Chandigarh, India. The ketamine–xylazine cocktail (1 mL of ketamine containing 100 mg/mL and 0.5 mL of xylazine containing 20 mg/mL and 8.5 mL saline) of 0.1 mL/10 g was used for anesthesia to minimize the pain of injection, and thrombophob cream was applied on the site of injection. Mice had free access to drinking water and food. Mice were monitored twice daily for clinical symptoms of infection or discomfort and euthanized if reaching humane endpoints specified in the ethical permissions. The experiment consisted of six treatment groups and six mice in each group. The mice were inoculated with 50 microliters of the optimum dose (1 × 10^8^ CFU/mL) via urethral catheterization, as previously described [14,23]. At 24 h and 48 h post-infection, groups of mice were treated twice a day subcutaneously with heraclenol at three doses (at MIC concentration, one concentration above MIC, and one below MIC) formulated in NaCl (0.9%). Urine samples were sampled for colony counts. On day one (the inoculum control group, PBS group, and drug-only group) and day three post-infection (bacterial infection followed by drug), mice were sacrificed after urine sampling by cervical dislocation. The bladder and kidneys were collected, weighed, and stored at −80 °C and later homogenized in one ml saline [24]. All samples were 10-fold diluted in saline, and 20 µL spots were applied on agar plates in duplicates, which were incubated for 18–22 h at 37 °C in Bio-Oxygen Demand (BOD) Incubator. CFU counts were normalized to milliliters and grams for urine and tissue samples, respectively. A median number of CFU/g or milliliters is indicated in the results. 

### 2.5. Histopathological Analysis

Kidney and bladder tissues were fixed in 10% buffered normal saline and dehydrated in gradient ethanol (30–100%). Paraffin wax blocks were prepared, and thin sections were stained by hematoxylin and eosin (H&E) staining [25]. The tissues were examined by a trained pathologist.

### 2.6. Biofilm Formation and Treatment

The biofilms were prepared by inoculating wells of 96 well microtiter plates with 200 µL of 24 h old liquid culture containing 10^8^ CFU/mL. Following 4 h of cell adhesion at 37 °C, the supernatant was removed from each well, and plates were rinsed with physiological saline. Subsequently, 200 µL of fresh medium containing heraclenol (at MIC value) was added to each well, and plates were further incubated for 24 h. Inoculated growth medium without heraclenol served as a positive control, and a growth medium supplemented with heraclenol was used as a negative control. The inhibition of biofilm formation was examined by the crystal violet staining method [26]. Briefly, after 24 h treatment, the supernatant was removed, and the well was rinsed with physiological saline. For the fixation of biofilm, methanol was added. After fixation, washing was done to remove extra fixatives. After that, a 0.1% crystal violet solution was added to each well and incubated at room temperature for 30 min. The excess dye was removed by washing the plates under running water gently. Finally, bound crystal violet was solubilized by adding 33% acetic acid, and the absorbance was measured at 590 nm.

### 2.7. Scanning Electron Microscopy (SEM)

The anti-biofilm effect of the heraclenol was also examined by SEM. For biofilm formation, 5 mL of an overnight culture of *E. coli* was added to the wells of 6-well microtiter plates. Sterile coverslips were placed in the wells, which served as attaching surfaces for the cells. The plates were incubated for 4 h at 37 °C; after that, the supernatant was removed, and the plates were rinsed with physiological saline. For the treatment of biofilm, 5 mL of drug solution (at MIC concentration) was added, and the well without the drug was used as an untreated control. A negative control sample contained only liquid culture media. Microtiter plates were incubated for 24 h. After incubation, the supernatant was removed, coverslips were washed with physiological saline and fixed with 2.5% formaldehyde, and dehydrated using a series of different concentrations of ethanol. After dehydration, coverslips were coated with the platinum membrane and then visualized using JSM-IT 300 scanning electron microscope [26]. 

### 2.8. Molecular Docking Studies for Analysis of Binding between Heraclenol and the HisC

The interaction between the heraclenol and the HisC enzyme was studied by using AutoDock [27]. Three-dimensional structural coordinates of the HisC were obtained from the Research Collaboratory for Structural Bioinformatics—Protein Data Bank (RCSB-PDB). The 2D structure of heraclenol was recovered in .sdf format from the ZINC database. Heraclenol structure was converted from .sdf format to .pdb by using Open babel software [28]. The crystal structure of the HisC of *E. coli* with a resolution of 1.5 Å was retrieved from the RCSB in PDB format with PDB ID 1fg7 [29,30]. The crystal structure downloaded from RCSB was not suitable for docking in its native form as it was crystallized with its cofactor PLP and substrate along with the water molecules. All the heteroatoms (water and ligand) of the molecule were deleted from the crystal structure using the protein preparation wizard embedded in the “AutoDock” [27]. Using a Lamarckian genetic process, heraclenol was docked into the HisC active site, a grid box was prepared and binding energy was computed. Similarly, the native substrate LHP and already known inhibitor semicarbazide were also docked to the protein’s active site and compared with heraclenol.

### 2.9. Statistical Analysis

One-way ANOVA and Dunnett’s multiple comparison tests were performed using the software GraphPad Prism 5. Differences were considered significant at a *p*-value of ≤0.05.

## 3. Results

**Figure 2:**
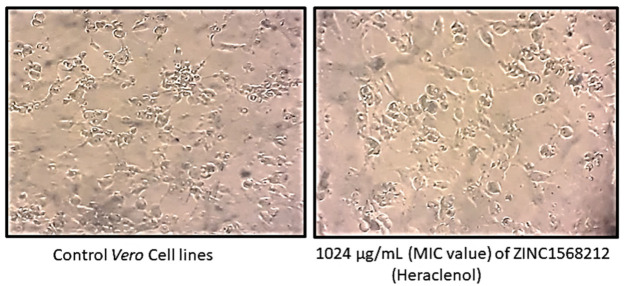
Effect of heraclenol on *Vero* cell lines. Heraclenol did not show any significant cytotoxicity as the morphology of the *Vero* cell line remained similar to the control cell line.

The MIC of the heraclenol was observed to be 1024 µg/mL. For the MTT assay, the results were expressed as the percentage viability (treated/control (non-treated) ×100) (Table 1). Heraclenol did not show any significant cytotoxicity as the morphology of the *Vero* cell line remained similar to the control cell line (Figure 2). There was only a 13% decrease in the cell viability at MIC value (Table 1).

### 3.1. Antibacterial Activity of Heraclenol In Vivo

**Figure 3:**
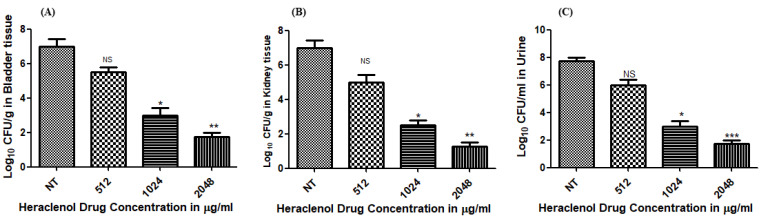
Heraclenol effectiveness in the clearance of UPEC infection from the bladder (**A**), kidney (**B**), and urine (**C**) in the in vivo mouse model. The statistical analysis was performed by using one-way ANOVA and Dunnett’s multiple comparison tests, * *p*, 0.05; ** *p*, 0.01; *** *p*, 0.001; NT—not tested (control), NS—nonsignificant.

We observed that heraclenol was effective at MIC and above MIC concentrations in clearing the infection from the urine, bladder, and kidney tissue (Figure 3).

### 3.2. Histopathology Analysis of Kidney Tissue

**Figure 4:**
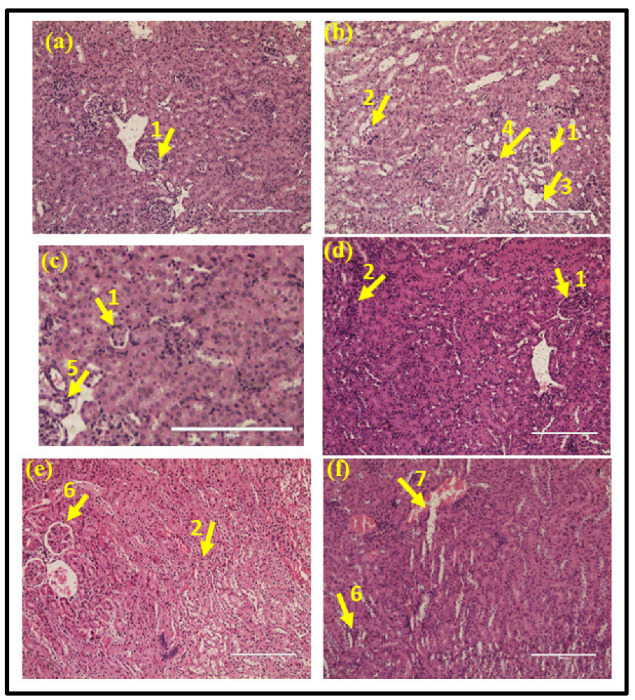
Histopathology analysis of kidney tissue: (**a**) normal uninfected mice (PBS), (**b**) infection control, (**c**) drug-only control (**d**) heraclenol treatment group at below MIC, (**e**) heraclenol treatment group at MIC value, (**f**) heraclenol treatment group at above MIC. 1: inflammation, 2: congestion, 3: dilation of Bowman’s capsule, 4: glomeruli congestion, 5: loss of tubular structure, 6: normal glomeruli, and 7: chronic inflammation.

Kidney tissues from uninfected mice and drug-only controls showed normal glomeruli (Figure 4a,c), whereas, in the mice infected with UPEC, signs of severe inflammation, congestion, dilation of Bowman’s capsule, and widespread infiltration of neutrophils were observed (Figure 4b). When compared, the infected mice treated with a below MIC value showed mild inflammation and congestion (Figure 4d). In the MIC group, little vascular congestion was seen, and glomeruli looked normal (Figure 4e). Similarly, infected mice treated with the above MIC value of the drug heraclenol showed normal glomeruli with mild chronic inflammation (Figure 4f). 

### 3.3. Histopathology Analysis of the Bladder Tissue

**Figure 5:**
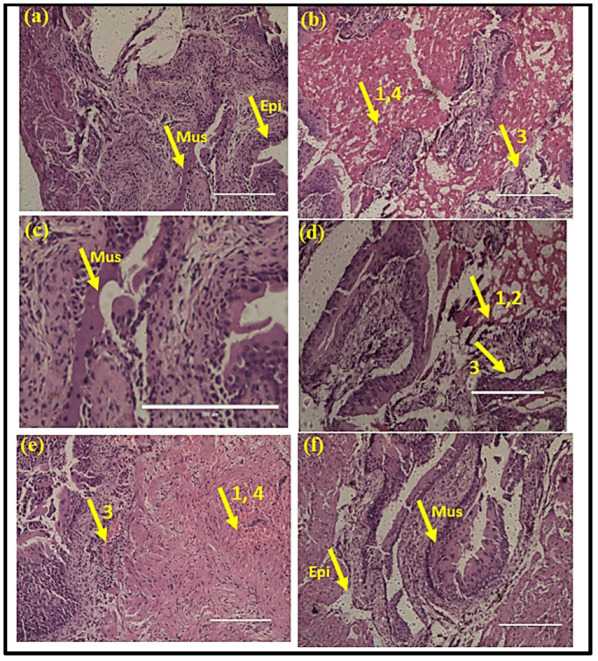
Histopathology analysis of bladder tissue: (**a**) normal uninfected mice (PBS), (**b**) infection control, (**c**) drug-only control, (**d**) heraclenol treatment (Below MIC), (**e**) heraclenol treatment (MIC), (**f**) heraclenol treatment (above MIC). 1: inflammation, 2: abrasion, 3: congestion, 4: hemorrhage, Mus: muscularis, Epi: epithelium.

The histological section of the uninfected mice showed a normal structure of the urinary bladder with intact translational epithelium, mucosa, and muscularis (Figure 5a). In the infected mice, the increased infiltration of neutrophils was observed in the bladder wall. A hemorrhage was also observed (Figure 5b). Similarly, in the drug-only control group, a normal structure of the urinary bladder was observed (Figure 5c). In contrast, in the below MIC value treatment group, moderate inflammation and congestion were observed (Figure 5d). In the MIC treatment group, mild chronic inflammation was observed that was focal in nature (Figure 5e). The histological section of the urinary bladder in the above MIC treatment group showed a normal urinary bladder with mild congestion (Figure 5f).

### 3.4. Antibiofilm Activity of Heraclenol

**Figure 6:**
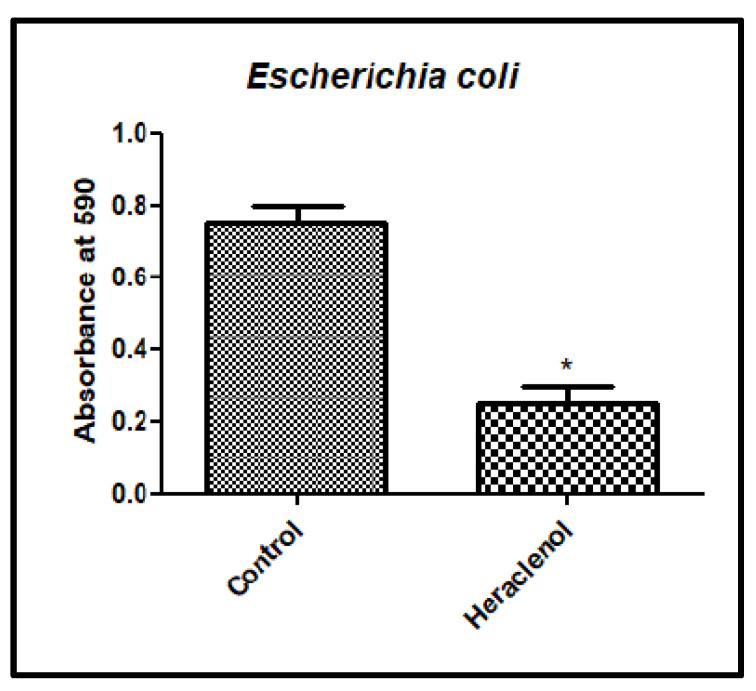
Heraclenol’s effect on biofilm formation. Biofilms were stained with crystal violet. * *p*, 0.05; comparison by Student’s *t*-test in Graph pad prism.

**Figure 7:**
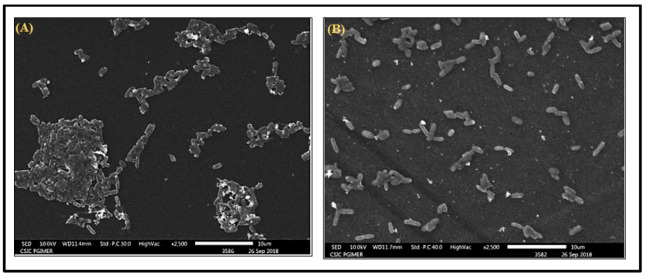
Scanning electron microscopic images of *Escherichia coli* MTCC 739 biofilms: (**A**) 24 h old control samples and (**B**) treated with heraclenol. Heraclenol was used at MIC value.

In the crystal violet assay, heraclenol reduced the development of biofilm by 70% (Figure 6). SEM results also showed that heraclenol had an inhibitory effect on the formation of biofilms. After treatment with heraclenol, there was a loss of cell-to-cell contact and decreased EPS production (Figure 7).

### 3.5. Interactional Analysis of HisC (Receptor) and Heraclenol (Ligand)

**Figure 8:**
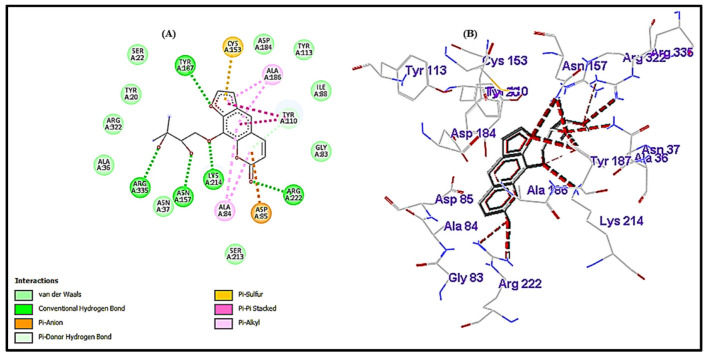
(**A**) The 2D plot of the interaction of heraclenol and HisC; hydrogen bonds are shown in green color lines. (**B**) Wireframe model of HisC with the heraclenol; red dotted lines represent the hydrogen bonds.

The interaction between the HisC (target) and the drug heraclenol was investigated using AutoDock and compared in terms of binding energy and hydrogen bond formation with the native substrate (LHP) and semicarbazide, a known inhibitor of HisC. From Figure 8, it can be observed that heraclenol formed five hydrogen bonds with TYR A: 187, ARG A: 222, LYS A: 214, ASN A: 157, and ARG A: 335 with a binding energy of −7.55 kcal/mol. Semicarbazide and LHP formed five and three hydrogen bonds, respectively. The binding energies of LHP and semicarbazide were −5.94 kcal/mol and −4.52 kcal/mol. Since heraclenol has the lowest binding energy, it can prove to be a very promising histidine biosynthesis inhibitor.

## 4. Discussion

In the present study, we demonstrated the antimicrobial and antibiofilm activity of heraclenol, a natural coumarin, for the first time against referenced invasive and biofilm-forming strains of UPEC. Heraclenol has been studied earlier and exhibited antibacterial activity against various pathogens, such as *S. aureus*, *S. epidermidis*, *P. aeruginosa*, *E. cloacae*, *K. pneumoniae*, *S. mutans*, and *S. viridans*, with MIC values of 0.68 mg/mL, 0.64 mg/mL, 0.70 mg/mL, 0.77 mg/mL, 0.85 mg/mL, 0.53 mg/mL, and 0.50 mg/mL, respectively [18]. In our study, heraclenol was observed to be effective at a concentration of 1024 µg/mL against UPEC strain CFT073, which is higher than earlier reported MICs. The difference in MIC obtained in our study and those obtained in the previous studies might be due to the highly virulent and MDR nature of the UPEC strain, CFT073 [31,32]. Even though our MICs were higher than earlier published reports, the in vivo efficacy results in clearing UTIs in the transurethral catheter mouse model were promising. Heraclenol significantly reduced bacterial loads at MIC and above MIC from urine, kidney, and bladder tissues. Histopathology analysis also complemented these results. Heraclenol restored the normal morphology of the bladder at 2048 µg/mL (above MIC concentration). Since the MIC of heraclenol is very high and is difficult to achieve clinically in tissues, it may either be used in lower doses in combination with other antibiotics for the treatment of UPEC infections, or chemical modifications may be required to boost its potency.

The antibacterial activity of heraclenol might be due to the presence of a coumarin ring, which has been known to inhibit the synthesis of nucleic acids in bacteria [33]. The addition of the prenyl group to the furocoumarins has been shown to enhance the lipophilicity of the molecule, and thus promote the entry of the molecules through the thick peptidoglycan layer of the bacterial cells [18]. The naturally produced coumarins are different from those of their parent molecule in terms of different substitutions, and the pyrano and furocoumarins are the most pharmacologically active molecules [34]. Parent coumarins showed comparatively lesser antibacterial activity than their substituted derivatives [35]. Bergamottin and dihydroxy-bergamottin, two furocoumarins isolated from grape juice have been shown to have anti-quorum sensing and antibiofilm potential in *E. coli* O157: H7 up to a magnitude of 72 and 58.3%, respectively [36]. These compounds also showed modest antibiofilm activity against other pathogens, such as *P. aeruginosa* and *S. enterica* serovar *typhimurium* [36]. We tested the in vitro antibiofilm activity of heraclenol using *E.coli* MTCC 739, which is a hyper biofilm-forming strain [37]. Heraclenol reduced the formation of biofilm by 70% in comparison to the control. Further, scanning electron microscopy results showed that heraclenol decreased the synthesis of extracellular matrix and cell-to-cell adhesion, which are hallmarks of biofilm formation. 

We validated the inhibition of histidine biosynthesis by studying in silico molecular interactions between the histidine biosynthesis enzyme HisC and heraclenol. By comparing its binding energy to that of the native substrate LHP, molecular docking studies showed that it binds to the HisC ligand-binding domain (LBD) very efficiently. The binding of heraclenol to the active site residues might block the conversion of imidazole-acetol-phosphate to histidinol-phosphate, an important reaction involved in the biosynthesis of histidine. Therefore, if we compare in terms of hydrogen bond formation and binding energy, heraclenol can prove to be a very significant protein biosynthesis inhibitor. Heraclenol showed minimal cytotoxicity in our study at all tested concentrations. At the MIC value, only a 13% decrease in cell viability was observed. This finding is in accordance with studies that demonstrated that aerial parts of the source plant *Ducrosia anethfolia* were non-toxic [38]. Site-directed mutagenesis experiments may be needed to confirm its exact mechanism of action. 

## 5. Conclusions

In conclusion, for the first time, we have demonstrated the antimicrobial and antibiofilm activity of heraclenol, a furocoumarin natural compound against UPEC. The drug reduced the bacterial load in the mouse UTI model by ≥4 logs and displayed antibiofilm activity. However, the MIC of heraclenol (1024 µg/mL) is high and not achievable clinically in human tissues. Therefore, further chemical modifications will be required to lower the drug’s MIC value and increase its potency. Alternatively, its synergistic action with other antimicrobials may also be studied. 

## Figures and Tables

**Table 1:** In vitro cytotoxicity analysis of heraclenol.

Sr. No.	Concentration (µg/mL)	Absorbance 595 nm	% Cell Viability
1	4096	0.7681	87.67
2	2048	0.7603	86.78
3	1024	0.7675	87.60
4	512	0.8253	94.20
5	Control Cell	0.8761	100

## Data Availability

Not applicable.
